# Thrombospondin-2 promotes the proliferation and migration of glioma cells and contributes to the progression of glioma

**DOI:** 10.1186/s41016-022-00308-x

**Published:** 2022-12-07

**Authors:** Tian-Lan Huang, Yi-Wen Mei, Yang Li, Xin Chen, Si-Xun Yu, Yong-Qin Kuang, Hai-Feng Shu

**Affiliations:** 1 Department of Neurosurgery, General Hospital of Western Theater Command of PLA, No.270 Rongdu Road, Jinniu District, 610083 Chengdu, China; 2 College of Medicine, Southwest Jiaotong University, No. 111, North Section 1, Second Ring Road, 610031 Chengdu, China

**Keywords:** Glioma, Migration, Proliferation, TSP2

## Abstract

**Background:**

Gliomas, especially high-grade gliomas, are highly malignant with a poor prognosis. Although existing treatments have improved the survival rate of patients with glioma, the recurrence and mortality rates are still not ideal. The molecular mechanisms involved in the occurrence and development of glioma are still poorly understood. We previously reported that thrombospondin-2 (TSP2) expression was increased in tumor specimens from rat models, promoting excitatory synapse formation. However, little is known about the effect of TSP2 on the biological characteristics of glioma.

**Methods:**

Glioma and cerebral cortex tissues were collected from 33 patients, and the expression of TSP2 in them was analyzed. Next, the proliferation and migration of TSP2 on glioma cells were analyzed in vitro. At last, a glioma transplantation model was constructed to explore the growth of TSP2 on glioma *in vivo*.

**Results:**

The expression of TSP2 in surgical glioma specimens was increased compared to that in the normal cortex. Interestingly, the TSP2 protein level was higher in high-grade glioma (HGG, World Health Organization (WHO) grades 3–4) than in low-grade glioma (LGG, WHO grades 1–2) tissues. Exogenous addition of the TSP2 protein at an appropriate concentration promoted the migration of glioma cells but did not significantly affect their proliferation. Surprisingly, overexpression of TSP2 promoted both the migration and proliferation of cultured glioma cells. Moreover, *in vivo* experimental data implied that overexpression of TSP2 in C6 cells promoted the malignant growth of gliomas, while knockout of TSP2 slowed glioma growth.

**Conclusions:**

TSP2 promotes the migration and proliferation of glioma cells, which may provide new ideas for blocking glioma progression.

**Supplementary Information:**

The online version contains supplementary material available at 10.1186/s41016-022-00308-x.

## Background

Glioma is a tumor derived from glial cells in the brain, and it is the most common primary tumor in the central nervous system (CNS) [1, 2]. According to the World Health Organization (WHO) classification system, glioma is histologically divided into low-grade glioma (LGG, WHO grades 1–2) and high-grade glioma (HGG, WHO grades 3–4) [3–5]. The statistical report of the Central Brain Tumor Registry of the United States (CBTRUS) indicates that glioblastoma has the highest incidence among HGG; moreover, glioblastoma has an extremely poor prognosis, with a 5-year survival rate of only 6.8% [6]. Although the survival of patients with glioma has been improved by surgery, immunotherapy, directed radiotherapy, chemotherapy and other treatment methods, the recurrence rate and mortality rate are still very high [7]. Glioma cells, which exhibit multiple and heterogeneous molecular alterations, migrate and invade by secreting extracellular matrix (ECM) molecules [8]. However, the mechanism of action of many molecules in the glioma microenvironment remains unclear.

Thrombospondin (TSP) is an adhesion protein that interacts with cells, and it is a family of secreted, multidomain glycoproteins that function on the cell surface and in the ECM environment [9–11]. TSP not only exerts oncogenic effects but also possesses tumor-suppressing properties and is considered an important component of the ECM [12]. Based on the characteristics of its molecular structure, TSP is classified into subgroup A (trimeric; TSP1 and TSP2) and subgroup B (pentameric; TSP3, TSP4, and TSP5) [13–15]. These proteins are not heteromeric but rather multimers of the same polypeptides. Proteins in subgroup A and subgroup B are both composed of an N-terminal domain (THBS-N), oligomerization domain, and a characteristic domain containing three epidermal growth factor (EGF)-like repeats, calcium-binding wire, and lectin-like C-terminal spheres [9, 13, 16]. In addition, proteins in subgroup A also contain a von Willebrand factor type C (VWC) domain, and three properdin-like repeats or thrombospondin repeats (TSRs) [9, 13, 16]. TSP has been reported to play a multifaceted role in the tumor microenvironment through which it regulates tumor progression. For example, TSP1 exerts an inhibitory effect on the growth of melanoma [17], TSP2 is associated with increased prostate cancer metastasis [18], TSP3 is associated with poor prognosis in osteosarcoma [19], and TSP4 is related to tumor suppression in colorectal cancer [20].

TSP1 and TSP2 have the same domains, and both have similar functions, such as inhibiting angiogenesis [16, 21], which plays an important role in cancer growth and development. Although evidence indicates that TSP1 and TSP2 are upregulated in tumors and play antiangiogenic roles leading to antitumor effects [22–24], the carcinogenic role of TSP1 that results in increased aggressiveness in glioma should not be ignored [25–28]. Similarly, as the TSP2 expression level varies with tumor type, its role in tumor progression remains controversial. Recent reports indicate that in the ECM-receptor interaction pathway, TSP2 is upregulated in some tumor tissues, playing an important role in tumor shedding, adhesion, degradation, movement, and proliferation [29]. For example, upregulated TSP2 is associated with a poor prognosis of lung cancer [30], prostate cancer [18], and oral cancer [31]. Surprisingly, the poor prognosis of ovarian cancer [32], cervical cancer [33], and gastric cancer [34] is related to the downregulation of TSP2. Despite these findings, the role of TSP2 in CNS tumors is still poorly understood.

The dataset mRNAseq_693 accessing from the Chinese Glioma Genome Atlas (CGGA, http://www.cgga.org.cn) was used to analyze the gene expression and prognosis of TSPs in glioma [35]. We found that TSP1, TSP2, TSP3, and TSP4 were all expressed in glioma, and the expression of TSP1, TSP2, TSP3, and TSP4 was statistically significant in different grades of glioma. Also, the survival rate of patients with low expression of TSP1, TSP2, and TSP4 was higher (Supplementary Fig. 1).

As shown in our previous report, glioma-derived TSP2 promotes excitatory synapse formation and results in hyperexcitability in the peritumoral cortex of glioma in a transplantation model, which may contribute to the occurrence of glioma-related epilepsy [36]. However, little is known about the effect of TSP2 on the biological characteristics of glioma itself. Accordingly, we hypothesized that TSP2 may be involved in regulating glioma growth, which is related to the clinical prognosis of patients. In this study, we examined the expression of TSP2 in surgical specimens from patients with glioma. We discussed the contribution of TSP2 to the proliferation and migration of glioma cells, and our findings may provide a new strategy for blocking the development of glioma.

## Methods

### Human specimens

We examined 28 surgical specimens from patients with LGG (WHO grade 2, *n* = 13, Supplementary Table 1) or HGG (WHO grades 3 or 4, *n* = 15, Supplementary Table 2). The diagnosis of glioma was confirmed by a neuropathological examination [5]. Normal-appearing cortex specimens obtained from patients with traumatic injuries were used for comparison (control, *n* = 5, Supplementary Table 3). These patients did not have a history of neurological diseases. All specimens were immediately frozen with liquid nitrogen after surgical resection and then stored in an ultralow temperature freezer (Sanyo, Japan) at −80 ℃ until subsequent use. Data on the patients were obtained from the databases compiled by the Neurosurgery Department of the General Hospital of Western Theater Command of PLA (China). All procedures and experiments were conducted according to the guidelines approved by the ethics committee of this hospital. All human specimens were used in a manner compliant with the Declaration of Helsinki.

### Western blot analysis

Glyceraldehyde 3-phosphate dehydrogenase (GAPDH) was selected as the loading control. Tissue was homogenized in protein extraction reagent (Keygen, China). The obtained homogenate was centrifuged at 12,000 rpm for 30 min at 4 °C. The protein samples collected from the supernatant were separated by sodium dodecyl sulfate–polyacrylamide gel electrophoresis (SDS-PAGE) at 8% concentration and transferred to 0.45-μm polyvinylidene difluoride membranes, which were subsequently blocked with TBST buffer for 2 h at 37 °C. The membranes were probed with primary rabbit anti-TSP1 (1:1000, MA5-13,398, Thermo Fisher Scientific, USA), rabbit anti-TSP2 (1:600, PA5-97,117, Thermo Fisher Scientific, USA), rabbit anti-TSP4 (1:1000, ab156258, Abcam, USA), and rabbit anti-GAPDH (1:2000, BA2913, Boster, USA) antibodies for 12 h at 4 °C. Next, the membranes were incubated with a horseradish peroxidase (HRP)-conjugated secondary goat anti-rabbit antibody (1:10,000, BA1054, Boster, USA) for 1 h at 37 °C. The immunoreactive bands were visualized with a chemiluminescent substrate (Thermo Fisher Scientific, USA). The optical densities (ODs) of the bands on the Western blots were quantified using Fiji software (USA). The relative expression levels of specific proteins were normalized and calculated as the optical density of the specific protein band/OD of the GAPDH band.

### Immunohistochemical (IHC) and immunofluorescence (IF) analyses

Tissues from surgical specimens, including tumor center tissues from patients with glioma and cerebral cortex tissues from patients with surgical injuries, were fixed with a 4% paraformaldehyde solution for 24 h at 4 °C. After dehydration and paraffin embedding, 7-μm-thick tissue sections were subjected to IHC staining using the avidin–biotin-peroxidase method. A primary rabbit anti-TSP2 antibody (1:200, GTX51797, GeneTex, USA) was diluted in 5% bovine serum albumin (BSA) in 0.01 M phosphate-buffered saline (PBS). Immunostaining was performed without primary antibodies as a negative control for each case.

IHC staining was performed in a double-blinded manner to assess the average optical density values. The TSP2 signal was quantified in randomly selected brain slices. The optical density was quantified in regions of interest in the tumor center of samples from patients with glioma and the cerebral cortex of patients with traumatic injuries. Three fields of view were quantified for each site. Semiautomated routines in Fiji were used to calculate the pixel area.

For IF staining, a Microm HM 525 Cryostat (Thermo Fisher Scientific, USA) was used to slice the fixed surgical specimen tissue into 35-μm sections. Primary antibodies, including a rabbit anti-TSP2 antibody (1:600, GTX51797, GeneTex, USA), rabbit anti-Iba1 antibody (1:1000, GTX100042, GeneTex, USA), mouse anti-GFAP antibody (1:600, ab10062, Abcam, UK), and mouse anti-NeuN antibody (1:100, GTX30773, GeneTex, USA), were diluted in 0.01 M PBS. FITC-conjugated goat anti-mouse (1:1000, bs-0296G-FITC, Bioss, China) and Cy3-conjugated goat anti-rabbit (1:300, GB21303, Servicebio, China) secondary antibodies were diluted in 0.01 M PBS. A Nikon A1R confocal microscope (Nikon, Japan) was used for image acquisition with consistent laser intensities and imaging settings. All compared images were acquired with the same exposure time.

### Cell culture

Human U251 glioma cells (U251) and human U87MG glioblastoma cells (U87MG) were obtained from Procell Life Science & Technology Co., Ltd (China). Rat C6 glioma cells (C6) were obtained from Cyagen Biosciences Inc. (China). All cells were cultured in high-glucose DMEM (HyClone, USA) supplemented with 10% fetal bovine serum (Gibco, USA) and a 1% penicillin–streptomycin solution (HyClone, USA) at 37 °C in a 5% CO_2_ atmosphere. All cells were used between passages 2 and 8 for experiments.

### Wound healing assay

Cells in logarithmic growth phase were detached with 0.25% trypsin–EDTA (Gibco, USA) to prepare cell suspensions. A total of 4 × 10^5^ cells were inoculated in 6-well plates and cultured at 37 °C in a 5% CO_2_ atmosphere until they adhered to the plate wall. A 10-μl pipette tip was used to create a vertical scratch in the cell layer after the confluence reached approximately 90%, and the medium was replaced with fetal bovine serum-free high-glucose DMEM. At the same time, the human TSP2 polypeptide (ab96113, Abcam, UK) was added. An optical microscope was used to acquire images at the marked points immediately and at different time points after the wound was created. The unhealed area was measured and analyzed with Fiji software to assess migration.

### Transwell (migration) assay

For the preparation of cell suspensions, cells in logarithmic growth phase were detached with 0.25% trypsin–EDTA after culture in serum-free medium for 24 h. Five-thousand cells were inoculated into the upper compartment of the transwell chamber, medium containing 10% fetal bovine serum was added to the lower compartment of the chamber, and the cells were incubated at 37 °C in a 5% CO_2_ atmosphere for 24 h. The human TSP2 polypeptide was added to the upper compartment of the transwell chamber. The cells on the upper surface of the transwell membrane were removed, and those on the lower surface were fixed with 4% paraformaldehyde for 10 min. After staining with crystal violet at room temperature for 15 min and rinsing with running water, the cells were counted under a microscope.

### Cell Counting Kit-8 (CCK-8) assay

Cells (50 cells/μl, total volume of 100 μl) harvested during logarithmic growth phase were seeded in 96-well plates at 37 °C in a 5% CO_2_ atmosphere. After the cells adhered to the plate wall, the TSP2 protein was added to the experimental group. The medium was replaced with fresh medium containing 10% CCK-8 reagent after 24, 48, or 72 h. The OD value at 450 nm was measured using a microplate reader (Thermo Fisher Scientific, USA) after 60 min of incubation at 37 °C in a 5% CO_2_ atmosphere.

### TSP2 overexpression

A lentivirus expressing TSP2 (LV-EF1a > rat Thbs2-CMV > eGFP/T2A/Puro) was obtained from Cyagen Biosciences Inc. (China). The lentivirus (multiplicity of infection (MOI) = 10) was added to glioma cells, and stable cells were selected with 1.6 μg/ml puromycin (4–5 passages). TSP2 overexpression in the stable cells was verified using qPCR [36] (data not shown).

### TSP2 knockout

A total of 2 × 10^5^ glioma cells were seeded into six-well plates. The lenti-CRISPR/Cas9-eGFP-puro-TSP2 knockout construct (gRNA-A1: ACCTTTGACCTTGCCGCACGTGG, gRNA-A2: AGCTCTGCGTCATATAGCTTAGG) obtained from Cyagen Biosciences Inc. (China) was transduced into C6 cells. Stable TSP2 knockout was verified in C6 cells by DNA sequencing [36] (data not shown).

### Stereotactic implantation

Twenty-one-day-old healthy male Wistar rats (Chengdu Dossy Experimental Animals Co., Ltd., China) were selected for glioma cell implantation [36–39]. Rats were anesthetized by administering an intraperitoneal injection of 10% chloral hydrate at 0.3 ml/100 g, and the head was then fixed in a stereotactic frame (RWD, China). The skull was fully exposed, and a 1-mm-diameter hole (3 mm anterior to the bregma, 3 mm lateral to the sagittal suture) was drilled on the left side. C6 cells (4 × 10^6^ cells/ml, total volume of 10 μl) were injected into the cortex with a stereotactic device at a rate of 1 μl/min. The vertical injection depth was 3.0 mm. The needle was retained in place for 10 min and was then slowly pulled out vertically. After the hole was sealed with bone wax, the scalp was sutured, and the skin was disinfected. One week after the C6 cells were implanted, the rat glioma transplantation model was used for subsequent experiments. This study was approved by the Ethics Committee of the General Hospital of Western Theater Command of PLA and was conducted in accordance with the international animal care guidelines established by the Declaration of Helsinki. Every effort was made to relieve the pain and discomfort of the animals.

### MRI analysis

After rats were anesthetized and fixed in a 7-cm small animal coil (MR750 3.0 T, GE, USA), coronal and sagittal MRI scans were performed. The scanning parameters were set as follows: layer thickness, 2 mm; layer spacing, 2 mm; and FOV, 120 mm × 120 mm. For T1WI, a 0Ax T1-FSE sequence was used, where the TR/TE was 6.072 ms/597 ms; for T2WI, a 0Ax T2-FSE sequence was used, where the TR/TE was 123.9 ms/5000 ms. The MRI analysis and tumor volume calculations were performed using syngo fastView software (Siemens, Germany). The image of the largest level of the tumor was selected to measure the volume. The following formula (mm^3^) was used for the calculation: length × width × height × (π/6).

### Statistical analysis

All data are presented as the mean ± SEM values. SPSS 26.0 (IBM, Armonk, NY, USA) and GraphPad Prism 8.0 software (GraphPad Software Inc., La Jolla, CA, USA) were used for statistical analyses. ImageJ software was used to analyze the gray values of the Western blots, optical density values of IHC images, gray values of IF images, and areas of cell wounds. An unpaired *t*-test was used for comparisons between two groups, and ANOVA followed by the Bonferroni post hoc test was used for comparisons among three or more groups. *P* < 0.05 was considered statistically significant.

## Results

### Upregulated TSP2 is derived mainly from glioma

**Fig. 1:**
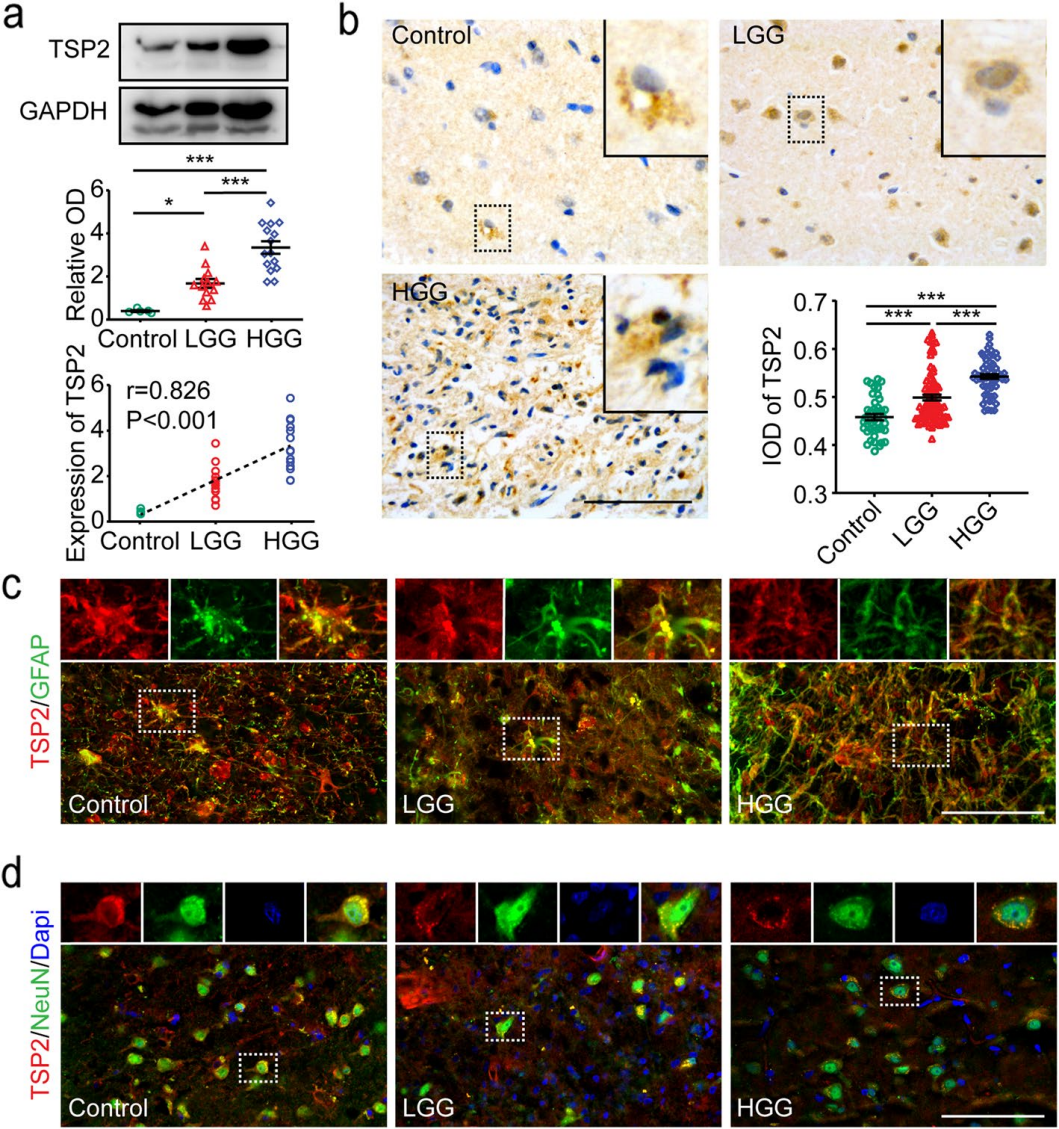
Expression and source of TSP2 in surgical specimens. **a** Representative Western blot bands (*upper panel*) showing TSP2 protein expression in the control group (control, *n* = 5), LGG group (*n* = 13), and HGG group (*n* = 15). Normalized densitometric analyses of Western blots (*middle panel*) showed a higher level of the TSP2 protein in the LGG (*P* = 0.028) and HGG groups (*P* < 0.001) than in the Ctrl group, and a higher level was detected in the HGG group than in the LGG group (*P* < 0.001). Spearman’s correlation analysis (*lower panel*) between the WHO grade of glioma and the expression of the TSP2 protein (*r* = 0.826, *P* < 0.001). **b** Representative IHC staining of surgical specimens from the center of LGGs/HGGs and the cerebral cortex of patients with traumatic injuries (control). The magnified view of each image shows the distribution of the TSP2 protein (bar: 100 μm). The average optical density values indicate higher expression of TSP2 in the LGG (*P* < 0.001) and HGG groups (*P* < 0.001) than in the Ctrl group and higher expression in the HGG group than in the LGG group (*P* < 0.001). **c** Representative IF staining for TSP2 (red) and GFAP (green) in the Ctrl group, LGG group, and HGG group (bar: 100 μm). The enlarged view of each image from left to right shows the TSP2 protein, GFAP ( +) cells, and the colocalization of TSP2 and GFAP. **d** Representative IF staining for TSP2 (red), NeuN (green), and DAPI (blue) in the Ctrl group, LGG group, and HGG group (bar: 100 μm). The enlarged view of each image from left to right shows the TSP2 protein, neurons, nuclei, and the colocalization of TSP2 and NeuN

Western blot analysis showed an increased TSP2 protein level in the tumor tissues from patients with LGG (*n* = 13, *P* = 0.028) and patients with HGG (*n* = 15, *P* < 0.001) compared with the control cortical tissue (*n* = 5, Fig. 1a). Notably, the TSP2 protein level in HGG tissues was even higher than that in LGG tissues (Fig. 1a, *P* < 0.001). Spearman’s correlation analysis showed a positive correlation between the expression of the TSP2 protein and the WHO grade of glioma (Fig. 1a, *r* = 0.826, *P* < 0.001) but no correlation with the age of patients (Supplementary Fig. 2, *r* =  − 0.122, *P* = 0.498). In addition, the TSP2 protein level in tissues from different genotypes (IDH wild type, IDH mutant, and NOS) was no statistical difference (*P* = 0.333). The semiquantitative IHC analysis further indicated that the TSP2 protein was highly concentrated in both LGG and HGG tissues and accumulated with increasing WHO grade (Fig. 1b). In addition, TSP2 was distributed mainly in the cytoplasm (Fig. 1b). We performed double immunolabeling of tissues from glioma tumor centers using an anti-TSP2 antibody to label the TSP2 protein, an anti-GFAP antibody to label reactive astrocytes, an anti-NeuN antibody to label neurons, and an anti-Iba1 antibody to label microglia to explore the source of the TSP2 protein. IF staining revealed the obvious colocalization of TSP2 and GFAP (Fig. 1c) and TSP2 and NeuN (Fig. 1d), but not TSP2 and Iba1 (Supplementary Fig. 3). Interestingly, the colocalization of TSP2 and NeuN was observed mainly at the edge of neuronal cells (Fig. 1d). Moreover, tissue from glioma tumor centers contained significantly fewer neurons than control tissue (data not shown). These findings implied that TSP2, which was secreted or accumulated in large amounts in tissues from the center of HGG, was derived mainly from reactive astrocytes or glioma cells and only partially from neurons.

### Exogenous TSP2 promotes glioma cell migration

**Fig. 2:**
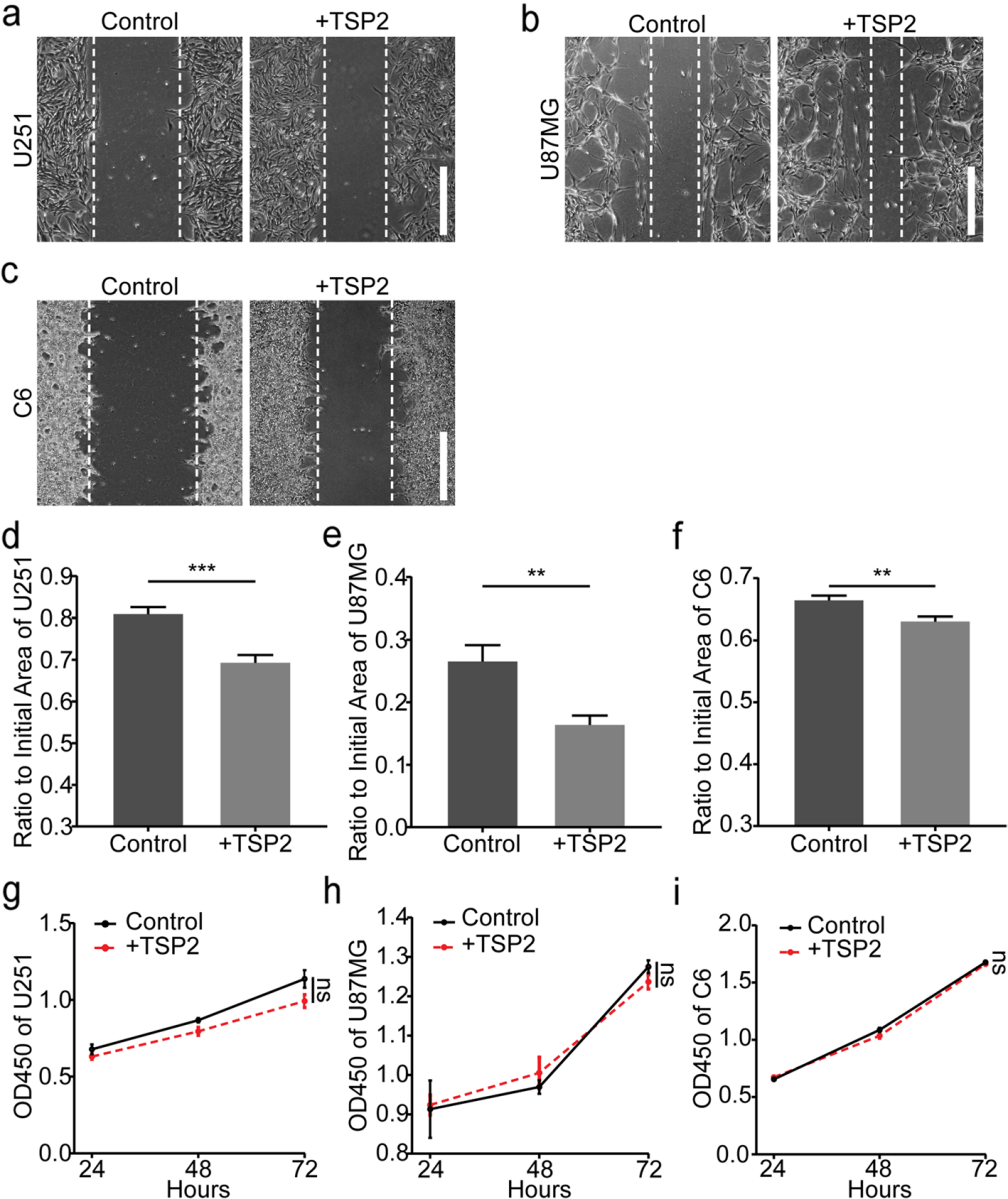
Effect of exogenous TSP2 on the migration and proliferation of glioma cells.** a–c** Representative images of scratch wounds of human U251 glioma cells, human U87MG glioblastoma cells, and rat C6 glioma cells after 48 h (bar: 500 μm). **d–f** Ratio of the unhealed area between 0 and 48 h after wounding in U251, U87MG, and C6 cells treated with or without the exogenous TSP2 protein. The group treated without the exogenous TSP2 protein was regarded as the control group. U251 cells (*P* < 0.001), U87MG cells (*P* = 0.002), and C6 cells (*P* = 0.001) treated with the exogenous TSP2 protein migrated faster at 48 h than those treated without the exogenous TSP2 protein. **g**–**i** In the CCK-8 assay, the optical density values at 450 nm showed that the proliferation rates of U251 (*P* = 0.126), U87MG (*P* = 0.501), and C6 (*P* = 0.076) cells treated with 0.72 μg/ml TSP2 protein were not significantly different from those of the corresponding cells not treated with exogenous TSP2 protein after 24, 48, or 72 h of culture (*P* = 0.126)

We added the exogenous TSP2 protein to glioma cells *in vitro* to explore its effect on their growth and the role of upregulated TSP2 in glioma. As shown in our previous studies, the most suitable concentration of TSP2 protein was 0.72 μg/ml; thus, we used this concentration in the present study. Cell wound healing assays showed that the addition of the exogenous TSP2 protein significantly promoted the migration of U251, U87MG, and C6 cells (Fig. 2 a–f). However, CCK-8 assays showed that the addition of the exogenous TSP2 protein did not promote the proliferation of U251, U87MG, and C6 cells (Fig. 2 g–i). In addition, even adding different concentrations of TSP2 protein in our previous studies did not promote the proliferation of U251 (*P* = 0.991, Supplementary Fig. 4) in our previous studies. Collectively, these findings indicate that the addition of an appropriate amount of the exogenous TSP2 protein promotes the migration, but not the proliferation, of glioma cells.

### TSP2 promotes glioma cell proliferation and migration

Viral transduction was used to overexpress the *rat TSP2* gene in C6 cells to generate cells overexpressing TSP2 (C6^TSP2+/+^) and to explore the effect of TSP2 on the biological characteristics of glioma cells. On the other hand, CRISPR/Cas technology was used to knock out the *rat TSP2* gene in C6 cells to generate cells lacking TSP2 expression (C6^TSP2−/−^). Wild-type C6 cells (C6^WT^) were used as control cells.

**Fig. 3:**
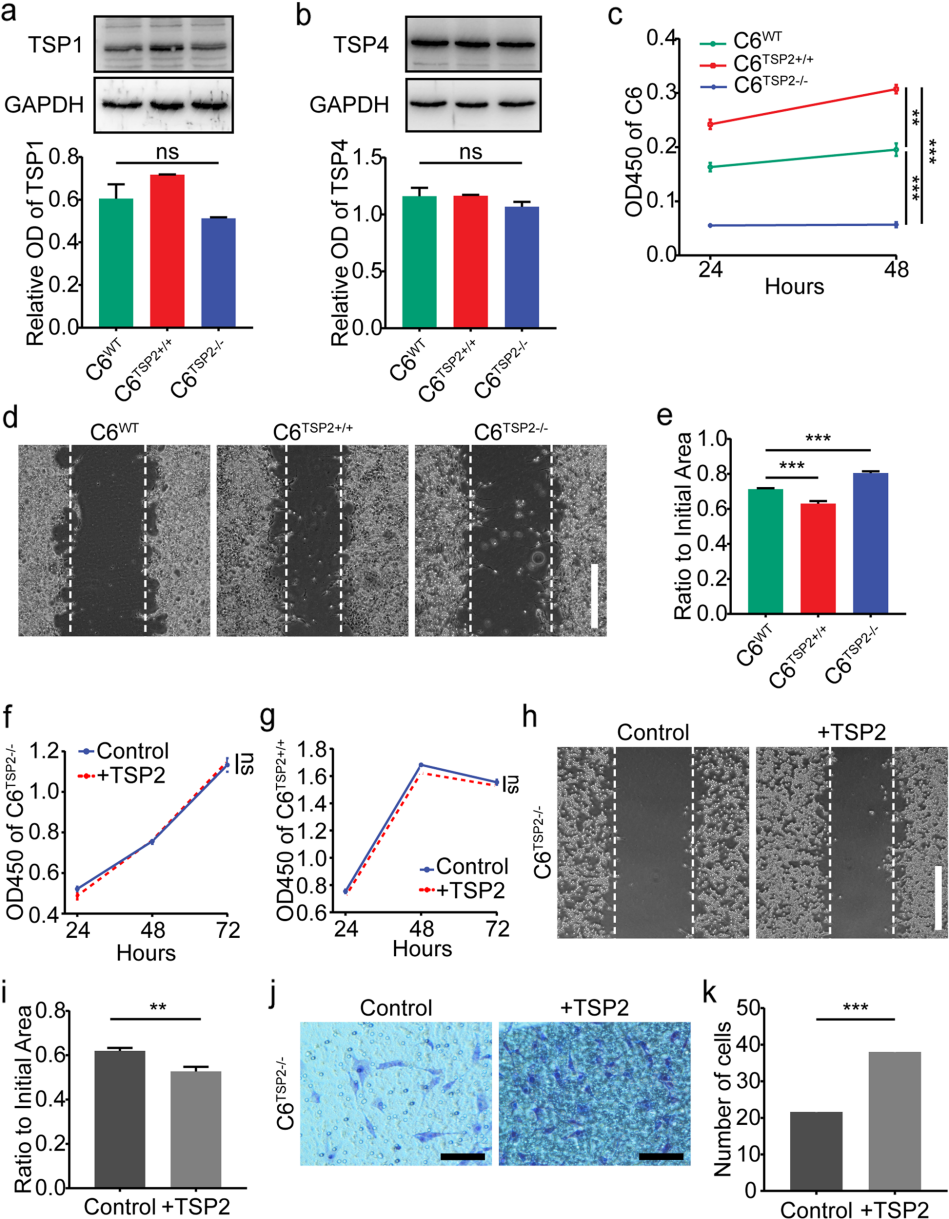
Effect of TSP2 protein expression on the migration and proliferation of glioma cells.** a–b** Western blot bands (*upper panels*) showing TSP1 and TSP4 protein expression in the C6^WT^, C6^TSP2+/+^, and C6^TSP2−/−^ groups. Experiments were repeated 3 times with cells at different passages. Densitometry analysis of Western blot bands (*lower panels*) showed that the TSP1 (C6^WT^ vs. C6^TSP2+/+^: *P* = 0.248, C6^WT^ vs. C6^TSP2−/−^: *P* = 0.344, C6^TSP2+/+^ vs. C6^TSP2−/−^: *P* = 0.067) and TSP4 (C6^WT^ vs. C6^TSP2+/+^: *P* = 0.999, C6^WT^ vs. C6^TSP2−/−^: *P* = 0.469, C6^TSP2+/+^ vs. C6^TSP2−/−^: *P* = 0.448) protein levels were not significantly different between the C6^WT^, C6^TSP2+/+^, and C6^TSP2−/−^ groups. **c** In the CCK-8 assay, the optical density values at 450 nm were recorded to determine the proliferation rates of C6^WT^, C6^TSP2+/+^, and C6^TSP2−/−^ cells after 24, 48, or 72 h of cultivation. Compared with C6^WT^ cells, the proliferation of C6^TSP2+/+^ cells was increased (*P* < 0.001), but the proliferation of C6^TSP2−/−^ cells was decreased (*P* < 0.001). **d** Representative images of scratch wounds of C6^WT^, C6^TSP2+/+^, and C6^TSP2−/−^ cells after 24 h (bar: 500 μm). **e** Ratio of the unhealed area between 0 and 24 h after wounding in C6^WT^, C6^TSP2+/+^, C6^TSP2−/−^ cells. C6^TSP2+/+^ cells (*P* < 0.001) migrated faster, and C6^TSP2−/−^ cells (*P* < 0.001) migrated slower than C6^WT^ cells at 24 h. **f**–**g** In the CCK-8 assay, the optical density values at 450 nm showed that the proliferation rates of C6^TSP2−/−^ cells and C6^TSP2+/+^ cells treated with the exogenous TSP2 protein were not significantly different from those were not treated with the exogenous TSP2 protein after 24, 48, and 72 h of culture (*P* = 0.174 and *P* = 0.551, respectively). **h** Representative images of scratch wounds of C6 cells after 48 h (bar: 500 μm). **i** Ratio of the unhealed area between 0 and 48 h after wounding in C6^TSP2−/−^ cells treated with or without the exogenous TSP2 protein. The group treated without the exogenous TSP2 protein was regarded as the control group. C6^TSP2−/−^ cells (*P* = 0.002) treated with the exogenous TSP2 protein migrated faster at 48 h than those that were not treated with the exogenous TSP2 protein. **j** Representative images showing the migration of C6^TSP2−/−^ cells cultured with or without the exogenous TSP2 protein in the chamber after 24 h (bar: 100 μm). **k** The number of migrated C6^TSP2−/−^ cells treated with the TSP2 protein was greater than the numbers of cells in the other groups after 24 h (*P* < 0.001)

Western blot analyses did not reveal significant difference in the expression of TSP1 and TSP4 in C6^TSP2+/+^ and C6^TSP2−/−^ cells compared with C6^WT^ cells (Fig. 3 a–b). Compared with the migration of C6^WT^ cells, the migration of C6^TSP2+/+^ cells was significantly increased, while that of C6^TSP2−/−^ cells was decreased (Fig. 3 d–e). Based on this finding, TSP2 significantly promoted the migration of C6 cells. Surprisingly, in the CCK-8 assay, the proliferation of C6^TSP2+/+^ cells was significantly increased, while the proliferation of C6^TSP2−/−^ cells was decreased compared with that of C6^WT^ cells, indicating that the TSP2 protein significantly increases the proliferation of C6 cells (Fig. 3c).

Next, we exogenously added the TSP2 protein to the culture environment of C6^TSP2−/−^ cells and found that it also promoted cell migration under these conditions (Fig. 3 h–i). The transwell assay yielded the same result (Fig. 3 j–k), indicating that the scratch wound assay was reliable. Combined with the results of previous experiments using C6^WT^ cells, these results further showed that exogenous TSP2 promotes the migration of glioma cells. However, exogenous TSP2 had almost no effect on the proliferation of C6^TSP2−/−^ cells and C6^TSP2+/+^ cells after addition to the cell culture environment (Fig. 3 f–j). This result further indicated that exogenous TSP2 does not affect the proliferation of glioma cells.

### TSP2 promotes the aggressive growth of glioma

**Fig. 4:**
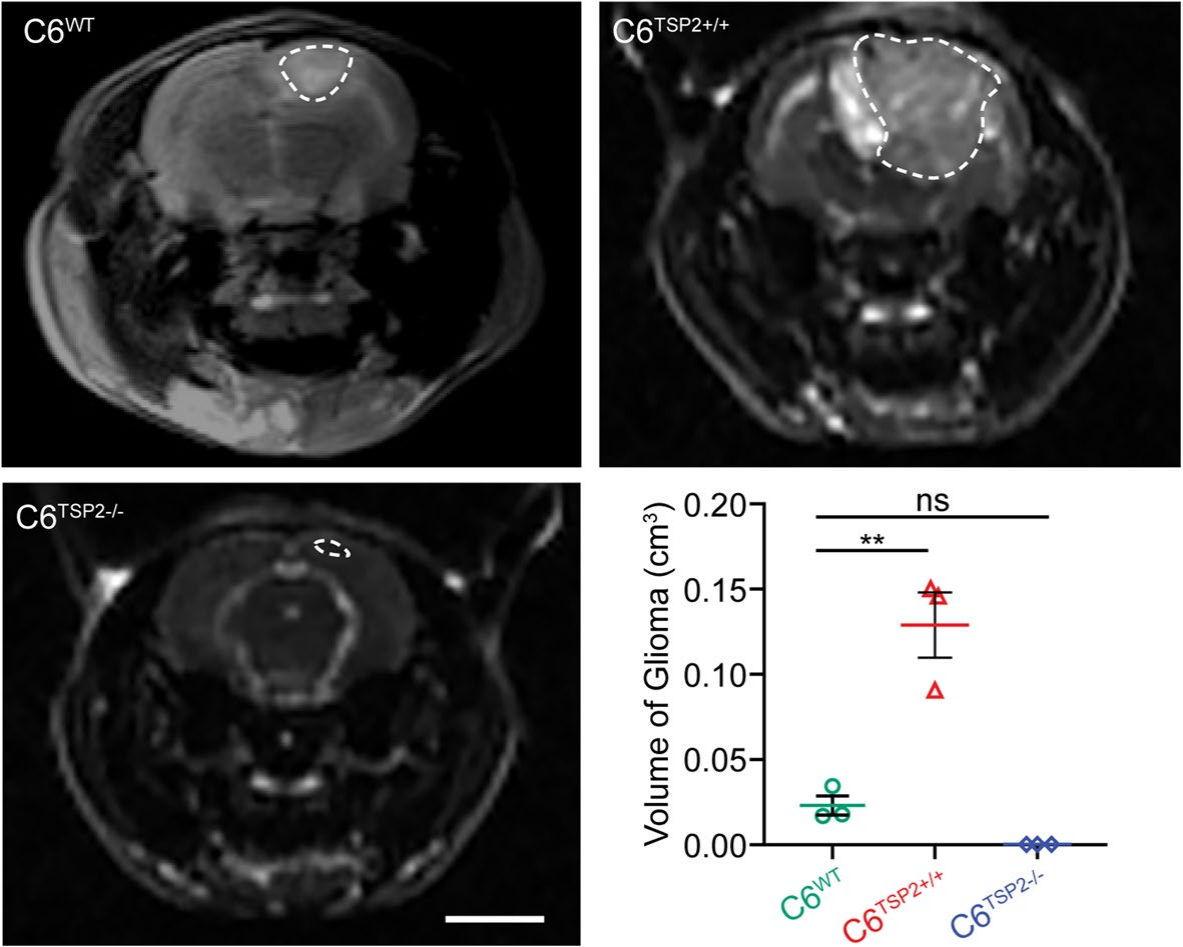
Characteristics of tumors in the glioma transplantation models. Representative T2WI MRI images of glioma transplantation models at the 3rd week of the implantation of C6^WT^ cells (C6^WT^ group, *n* = 3), C6^TSP2+/+^ cells (C6^TSP2+/+^ group, *n* = 3), and C6^TSP2−/−^ cells (C6^TSP2−/−^ group, *n* = 3) in the cerebral cortex of rats (bar: 5 mm). The locations of the tumors are indicated by the dashed boxes. The volume analysis revealed a large tumor volume in the C6^TSP2+/+^ group than that in the C6^WT^ group (*P* = 0.002), but they were not significantly different between the C6^TSP2−/−^ group and C6^WT^ group (*P* = 0.470)

We established a rat model of C6 glioma transplantation to study the effect of TSP2 on the growth of glioma in an environment where the synaptic structures adjacent to glioma were remodeled *in vivo* [36–40]. The MRI analysis showed that glioma grew aggressively in the rat brain, and that a peritumoral edema zone formed. The glioma tumor volume peaked for about the 3rd weeks after the transplantation of C6 cells into the rat cerebral cortex. The volume of glioma was measured, and the volume of glioma in the C6^TSP2+/+^ group was significantly larger than that in the C6^WT^ group (*P* = 0.037) at the 3rd week (Fig. 4). Moreover, the survival rate of rats in the C6^TSP2+/+^ transplantation model was lower than that of rats transplanted with C6^WT^ cells (Supplementary Fig. 5). Therefore, we speculated that TSP2 promoted the aggressive growth of glioma.

## Discussion

Based on the results of the present study, the expression of TSP2 in surgical glioma specimens was increased compared to that in the normal cortex. Interestingly, the TSP2 protein level was higher in HGG tissues than in LGG tissues, consistent with our previous report on animal transplantation models showing that TSP2 was derived mainly from the glioma center and only partially derived from reactive astrocytes [36] and neurons [41–43]. In addition, the expression of the TSP2 protein was positively correlated with the WHO grade of gliomas. Addition of the exogenous TSP2 protein at an appropriate concentration promoted the migration of U251, U87, and C6 cells, but it had no significant effect on their proliferation. Surprisingly, overexpression of TSP2 promoted both the migration and proliferation of glioma cells. Moreover, our *in vivo* experimental data implied that overexpression of TSP2 promoted the malignant growth of gliomas, while knockout of *TSP2* slowed glioma growth. Taken together, our data show that TSP2 promotes the migration and proliferation of glioma cells, which is closely related to the occurrence and development of glioma.

Our findings implied that the expression of the TSP2 protein was upregulated in gliomas, consistent with previous reports [44]. Moreover, TSP2 expression was higher in HGG than in LGG. TSP2 has a wide range of sources. In the present study, TSP2 was derived mainly from the glioma center and was only partially derived from GFAP-positive glial cells. Surprisingly, the IF data indicated that the glioma center contained a few neurons, which expressed the TSP2 protein. According to previous studies, the TSP2 protein is also expressed in rat retinal ganglion cells [42] and mouse nerve ganglion cells [41], and TSP2 mRNA expression was detected in adult nerve tissue and fetal brain nerve tissue [43]. As shown in our previous studies, reactive astrocytes accumulate around gliomas, which may also contribute to the pool of TSP2 [36].

A large number of studies have shown that TSP2 is related mainly to angiogenesis in malignant tumors. However, the biological effects of TSP2 on tumor cells are not understood. Therefore, we focused on the role of TSP2 in glioma cells in this study. After TSP2 was overexpressed or knocked out in C6 cells, the levels of its isoforms TSP1 and TSP4 did not undergo compensatory changes. Both exogenous application and endogenous overexpression of the TSP2 protein significantly promoted, but *TSP2* knockout inhibited the migration of glioma cells. Interestingly, the migration-promoting effect of TSP2 on U251 cells was achieved after the concentration of exogenous TSP2 was increased to a certain value (0.72 μg/ml). However, a higher concentration of TSP2 (greater than 0.72 μg/ml) did not promote cell migration. Recent evidence has shown that TSP2 induces vascular smooth muscle cell (VSMC) migration in a dose-dependent manner [45]. Accordingly, we speculated that TSP2 promotes the migration of glioma cells within a certain concentration range, but the detailed mechanism requires further study.

In addition, we found that TSP2 overexpression significantly promoted, but TSP2 knockout inhibited the proliferation of C6 glioma cells. However, exogenous addition of TSP2 protein did not seem to affect the proliferation of U251, U87MG, and C6 cells. The explanation for this differential effect on the proliferation of glioma may be that overexpression or knockout of TSP2 directly affects the cell division cycle. The transplantation model further illustrated the effect of TSP2 on the malignant growth of glioma *in vivo*. C6 cells with TSP2 overexpression and C6 cells with TSP2 knockout exhibited opposite growth patterns in the cerebral cortex of rats. Overexpression of TSP2 evidently promoted the malignant invasion and proliferation of glioma cells. Admittedly, there may be some limitations to employ non-transgenic animals, as the TSP2 may be partially secreted by non-transgenic animals themselves, which may have some impact on this study. Nevertheless, it can still be observed that the rat model of C6^TSP2−/−^ and C6^TSP2+/+^ glioma transplantation can also achieve some research significance. We previously reported that glioma-derived TSP2 leads to an increase in the number of excitatory synapses adjacent to the tumor, causing excessive excitation of the peritumoral cortex and participating in the occurrence of peritumoral epileptiform discharges in the transplantation model [36]. Recently, the imbalance of the neural network adjacent to glioma was reported to promote the progression of gliomas [46, 47]. Furthermore, combined with the results of the *in vitro* studies described above, we noticed that TSP2 may use two pathways to promote the proliferation of glioma cells in this study. On the one hand, the overexpression of TSP2 in glioma cells promotes the proliferation of glioma cells. On the other hand, the invasiveness of gliomas is enhanced, which is achieved by TSP2-mediated remodeling of synaptic structures adjacent to tumors. These results further confirm that TSP2 exhibits carcinogenic effects on glioma.

## Conclusion

This study provides evidence that TSP2 promotes tumor development. Specifically, TSP2 acts on gliomas to promote their development, on the one hand, via a mechanism related to the migration and proliferation of glioma cells. On the other hand, TSP2 may mediate the imbalance of the peritumoral cortex neural network to promote the biological progression of glioma. In this study, this biological role of TSP2 may be related to the prognosis of patients with glioma.

## Supplementary Information

**Additional file 1: Supplementary Fig. 1.** Expression and prognosis of TSPs in glioma.

**Additional file 2: Supplementary Fig. 2.** Analysis of the Spearman correlation coefficients between the expression of TSP2 protein and the age of patients.

**Additional file 3: Supplementary Fig. 3.** The source of the TSP2 protein in surgical specimens.

**Additional file 4: Supplementary Fig. 4.** Effects of different concentrations of TSP2 protein on the proliferation of U251 cells.

**Additional file 5: Supplementary Fig. 5.** The survival curves of rats after implantation of glioma cells showed that the survival rate in the C6^TSP2+/+^ group (*n*=3) was reduced compared with that in the C6^WT^ group (*n*=3).

**Additional file 6: Supplementary Table 1.** Clinical characteristics of patients with low grade glioma.

**Additional file 7: Supplementary Table 2.** Clinical characteristics of patients with High grade glioma.

**Additional file 8: Supplementary Table 3.** Characteristics of patients with traumatic brain injury for control group.

## Data Availability

Not applicable.

## Abbreviations

BSA: Bovine serum albumin
CCK-8: Cell Counting Kit-8
CNS: Central nervous system
ECM: Extracellular matrix
GAPDH: Glyceraldehyde 3-phosphate dehydrogenase
HGG: High-grade glioma
HRP: Horseradish peroxidase
IF: Immunofluorescence
IHC: Immunohistochemical
LGG: Low-grade glioma
MOI: Multiplicity of infection
ODs: Optical densities
PBS: Phosphate-buffered saline
SDS-PAGE: Sodium dodecyl sulfate–polyacrylamide gel electrophoresis
TSP: Thrombospondin
VSMC: Vascular smooth muscle cell
WHO: World Health Organization
