# Supplementary figures and images for: Thrombospondin-2 promotes the proliferation and migration of glioma cells and contributes to the progression of glioma
Source: Chin Neurosurg J. 2022 Dec 7;8:39. doi: 10.1186/s41016-022-00308-x (PMC9728004; doi:10.1186/s41016-022-00308-x)

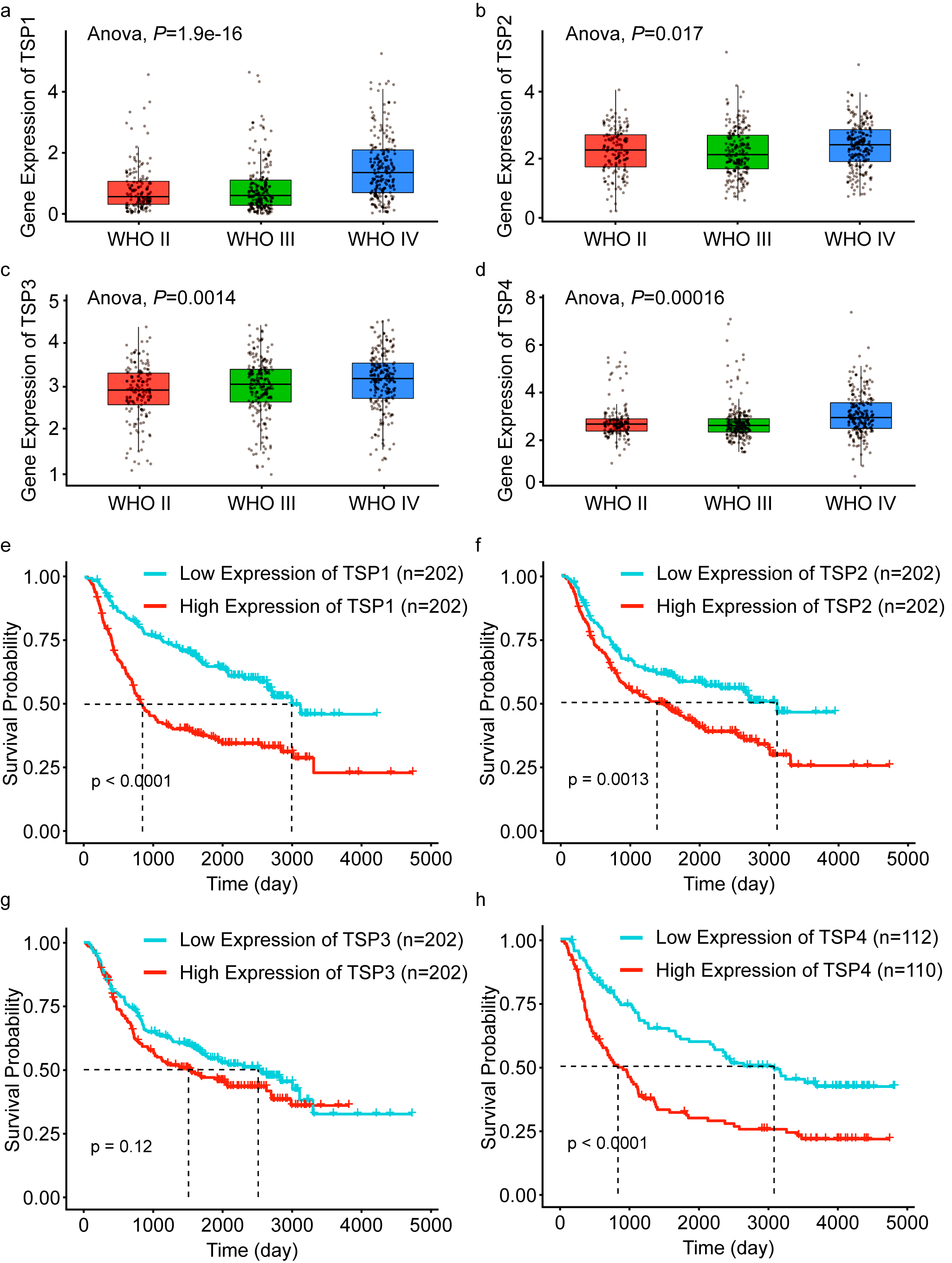

Supplement: Supplementary file 1 — Additional file 1: Supplementary Fig. 1. Expression and prognosis of TSPs in glioma. [file 41016_2022_308_MOESM1_ESM.tif]

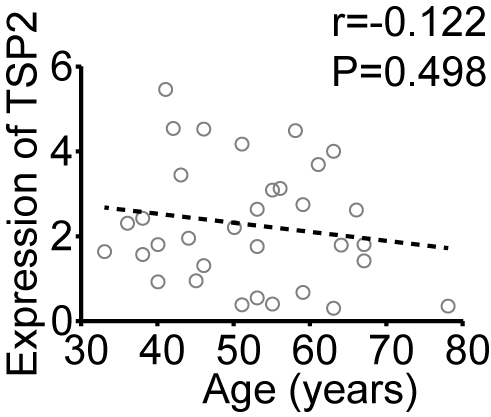

Supplement: Supplementary file 2 — Additional file 2: Supplementary Fig. 2. Analysis of the Spearman correlation coefficients between the expression of TSP2 protein and the age of patients. [file 41016_2022_308_MOESM2_ESM.tif]

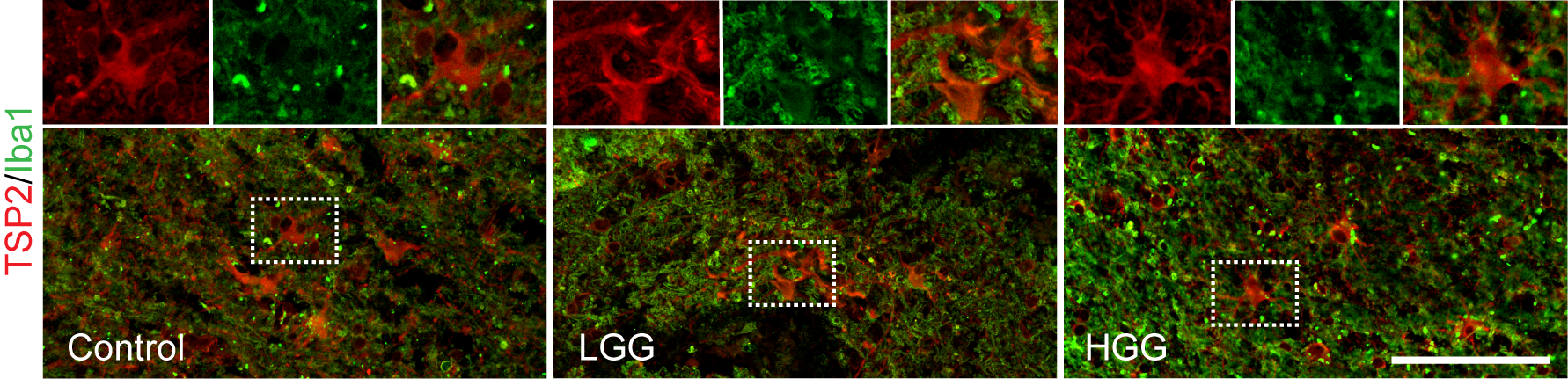

Supplement: Supplementary file 3 — Additional file 3: Supplementary Fig. 3. The source of the TSP2 protein in surgical specimens. [file 41016_2022_308_MOESM3_ESM.tif]

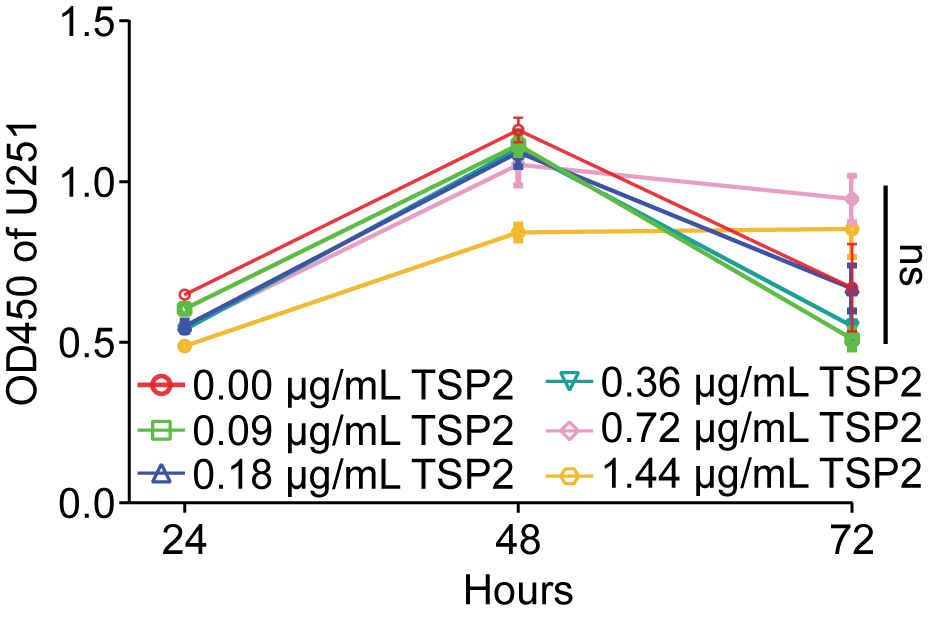

Supplement: Supplementary file 4 — Additional file 4: Supplementary Fig. 4. Effects of different concentrations of TSP2 protein on the proliferation of U251 cells. [file 41016_2022_308_MOESM4_ESM.tif]

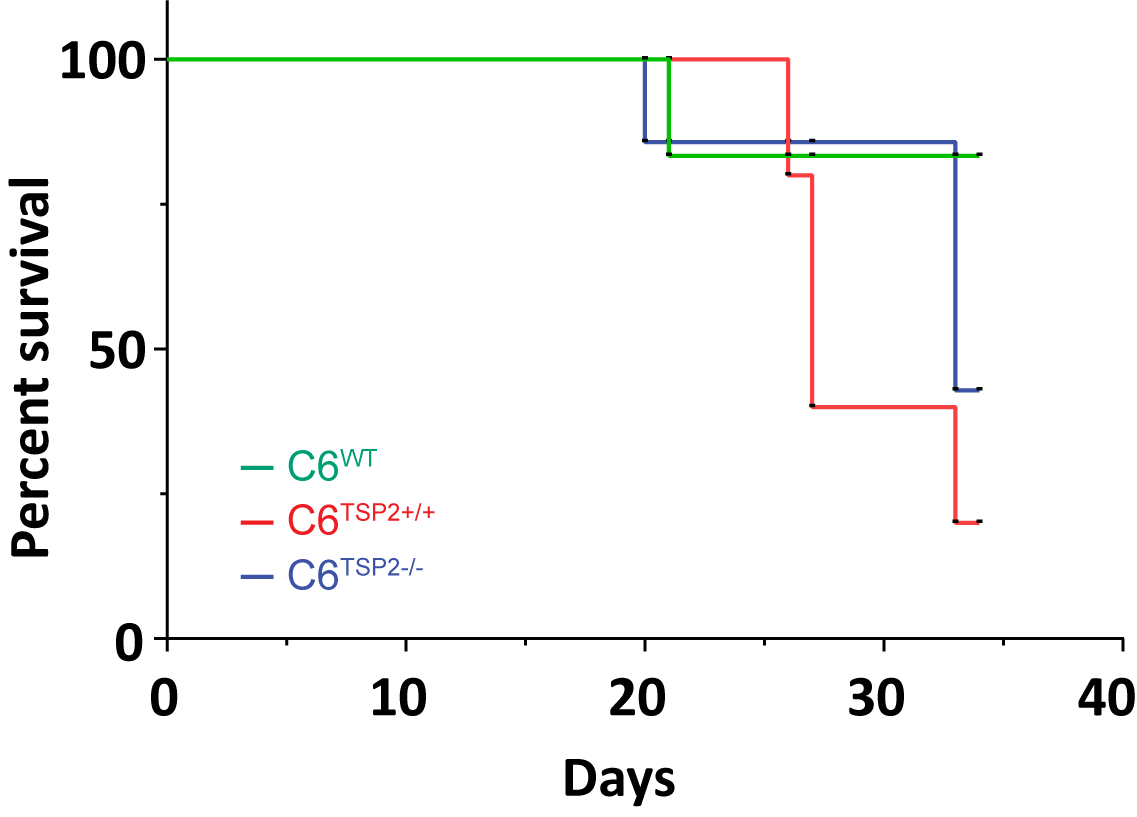

Supplement: Supplementary file 5 — Additional file 5: Supplementary Fig. 5. The survival curves of rats after implantation of glioma cells showed that the survival rate in the C6TSP2+/+ group (n=3) was reduced compared with that in the C6WT group (n=3). [file 41016_2022_308_MOESM5_ESM.tif]
