# Supplementary material for: Thrombospondin-2 promotes the proliferation and migration of glioma cells and contributes to the progression of glioma
Source: Chin Neurosurg J. 2022 Dec 7;8:39. doi: 10.1186/s41016-022-00308-x (PMC9728004; doi:10.1186/s41016-022-00308-x)
Supplement: Supplementary file 6 — Additional file 6: Supplementary Table 1. Clinical characteristics of patients with low grade glioma. [file 41016_2022_308_MOESM6_ESM.docx]

**Supplementary Table 1 Clinical characteristics of patients with low grade glioma**

| **Subject NO.** | **Sex** | **Age** | **Tumor location** | **Tumor type** | **Genetic information** | **Tumor grade** |
| --- | --- | --- | --- | --- | --- | --- |
| 1 | M | 40 | Right parietal lobe | astrocytoma, NOS | IDH1R132H (-) | WHO grade II |
| 2 | M | 38 | Right frontal lobe | astrocytoma, IDH-mutant | IDH1R132H (+) | WHO grade II |
| 3 | M | 53 | Left frontotemporal lobe | Diffuse astrocytoma, IDH-mutant | — | WHO grade II |
| 4 | M | 43 | Left temporal lobe | astrocytoma, NOS | — | WHO grade II |
| 5 | M | 50 | Left frontal parietal lobe | astrocytoma, NOS | — | WHO grade II |
| 6 | F | 53 | Right temporal insula lobe | Diffuse astrocytoma, IDH-mutant | IDH1R132H (+) | WHO grade II |
| 7 | F | 46 | Left frontal lobe | Diffuse astrocytoma, IDH-mutant | IDH1R132H (+) | WHO grade II |
| 8 | M | 40 | Left parietal lobe | astrocytoma, NOS | IDH1R132H (-) | WHO grade II |
| 9 | M | 33 | Right frontal lobe | Diffuse astrocytoma, NOS | — | WHO grade II |
| 10 | M | 59 | Left temporal parietal lobe | astrocytoma, NOS | — | WHO grade II |
| 11 | F | 44 | Left temporal lobe | Diffuse astrocytoma, IDH-wildtype | — | WHO grade II |
| 12 | M | 45 | Left frontal lobe | Diffuse astrocytoma, NOS | — | WHO grade II |
| 13 | F | 67 | Right frontal lobe | Diffuse astrocytoma, NOS | — | WHO grade II |

M=male; F=female; IDH: isocitrate dehydrogenase; NOS: not otherwise specified.
