# Supplementary material for: Thrombospondin-2 promotes the proliferation and migration of glioma cells and contributes to the progression of glioma
Source: Chin Neurosurg J. 2022 Dec 7;8:39. doi: 10.1186/s41016-022-00308-x (PMC9728004; doi:10.1186/s41016-022-00308-x)
Supplement: Supplementary file 7 — Additional file 7: Supplementary Table 2. Clinical characteristics of patients with High grade glioma. [file 41016_2022_308_MOESM7_ESM.docx]

**Supplementary Table 2 Clinical characteristics of patients with High grade glioma**

| **Subject NO.** | **Sex** | **Age** | **Tumor location** | **Tumor type** | **Genetic information** | **Tumor grade** |
| --- | --- | --- | --- | --- | --- | --- |
| 1 | F | 64 | Left frontal lobe | Glioblastoma, IDH-wildtype | IDH1R132H (-) | WHO grade IV |
| 2 | M | 38 | Right frontal lobe | Anaplastic astrocytoma, IDH-wildtype | — | WHO grade III |
| 3 | M | 46 | Right frontotemporal lobe | Anaplastic astrocytoma, IDH-mutant | IDH1R132H (+) | WHO grade III |
| 4 | M | 51 | Right temporal parietal lobe | Glioblastoma, IDH-mutant | IDH1R132H (+) | WHO grade IV |
| 5 | M | 58 | Right temporal parietal lobe | Glioblastoma, IDH-mutant | — | WHO grade IV |
| 6 | M | 41 | Right frontotemporal lobe | Glioblastoma, NOS | IDH1R132H (-) | WHO grade IV |
| 7 | M | 36 | Right parietal lobe | Glioblastoma, IDH-mutant | IDH1R132H (+) | WHO grade IV |
| 8 | M | 59 | Left temporal lobe | Glioblastoma, NOS | — | WHO grade IV |
| 9 | F | 67 | Right frontal lobe | Glioblastoma, IDH-mutant | IDH1R132H (+) | WHO grade IV |
| 10 | F | 56 | Right temporal lobe | Glioblastoma, IDH-mutant | IDH1R132H (+) | WHO grade IV |
| 11 | M | 42 | Left parietal lobe | Anaplastic astrocytoma, IDH-wildtype | — | WHO grade III |
| 12 | F | 63 | Right parietal lobe | Glioblastoma, IDH-mutant | IDH1R132H (+) | WHO grade IV |
| 13 | M | 61 | Left frontal lobe | Glioblastoma, IDH-wildtype | — | WHO grade IV |
| 14 | M | 66 | Left frontal lobe | Glioblastoma, IDH-mutant | IDH1R132H (+) | WHO grade IV |
| 15 | F | 55 | Right temporal lobe | Anaplastic astrocytoma, IDH-wildtype | IDH1R132H (-) | WHO grade III |

M=male; F=female; IDH: isocitrate dehydrogenase; NOS: not otherwise specified.
