# Supplementary material for: Thrombospondin-2 promotes the proliferation and migration of glioma cells and contributes to the progression of glioma
Source: Chin Neurosurg J. 2022 Dec 7;8:39. doi: 10.1186/s41016-022-00308-x (PMC9728004; doi:10.1186/s41016-022-00308-x)
Supplement: Supplementary file 8 — Additional file 8: Supplementary Table 3. Characteristics of patients with traumatic brain injury for control group. [file 41016_2022_308_MOESM8_ESM.docx]

**Supplementary Table 3 Characteristics of patients with traumatic brain injury for control group**

| **Subject No.** | **Sex** | **Age** | **Brain region** | **Diagnosis** | **History of glioma (YES/NO)** |
| --- | --- | --- | --- | --- | --- |
| 1 | F | 51 | Frontal lobe | Left frontal lobe trauma | NO |
| 2 | F | 63 | Frontal lobe | Right frontal lobe trauma | NO |
| 3 | M | 78 | Temporal lobe | Left frontal lobe trauma | NO |
| 4 | F | 53 | Temporal lobe | Right frontal lobe trauma | NO |
| 5 | M | 55 | Temporal lobe | Right frontal lobe trauma | NO |

M=male; F=female.
